# IL-1β Promotes Corneal Epithelial Cell Migration by Increasing MMP-9 Expression through NF-κB- and AP-1-Dependent Pathways

**DOI:** 10.1371/journal.pone.0057955

**Published:** 2013-03-07

**Authors:** Hui-Ching Tseng, I-Ta Lee, Chih-Chung Lin, Pei-Ling Chi, Shin-Ei Cheng, Ruey-Horng Shih, Li-Der Hsiao, Chuen-Mao Yang

**Affiliations:** 1 Department of Physiology and Pharmacology and Health Aging Research Center, College of Medicine, Chang Gung University, Kwei-San, Tao-Yuan, Taiwan; 2 Department of Anesthetics, Chang Gung Memorial Hospital at Linkou and College of Medicine, Chang Gung University, Kwei-San, Tao-Yuan, Taiwan; University Heart Center Freiburg, Germany

## Abstract

Interleukin-1β (IL-1β) plays a critical mediator in the pathogenesis of eye diseases. The implication of IL-1β in inflammatory responses has been shown to be mediated through up-regulation of inflammatory genes, including matrix metalloproteinase-9 (MMP-9). However, the detailed mechanisms of IL-1β-induced MMP-9 expression in Statens Seruminstitut Rabbit Corneal Cells (SIRCs) are largely unclear. Here, we demonstrated that in SIRCs, IL-1β induced MMP-9 promoter activity and mRNA expression associated with an increase in the secretion of pro-MMP-9. IL-1β-induced pro-MMP-9 expression and MMP-9 mRNA levels were attenuated by pretreatment with the inhibitor of MEK1/2 (U0126), JNK1/2 (SP600125), NF-κB (Bay11-7082), or AP-1 (Tanshinone IIA) and transfection with siRNA of p42 or JNK2. Moreover, IL-1β markedly stimulated p42/p44 MAPK and JNK1/2 phosphorylation in SIRCs. In addition, IL-1β also enhanced p42/p44 MAPK translocation from the cytosol into the nucleus. On the other hand, IL-1β induced c-Jun and c-Fos mRNA expression, c-Jun phosphorylation, and AP-1 promoter activity. NF-κB translocation, IκBα degradation, and NF-κB promoter activity were also enhanced by IL-1β. Pretreatment with U0126 or SP600125 inhibited IL-1β-induced AP-1 and NF-κB promoter activity, but not NF-κB translocation from the cytosol into the nucleus. Finally, we established that IL-1β could stimulate SIRCs migration via p42/p44 MAPK-, JNK1/2-, AP-1-, and NF-κB-dependent MMP-9 induction. These results suggested that NF-κB and AP-1 activated by JNK1/2 and p42/p44 MAPK cascade are involved in IL-1β-induced MMP-9 expression in SIRCs.

## Introduction

Dry eye disease is an extremely common ocular disorder, and large epidemiologic studies, using a variety of definitions, have estimated its prevalence at approximate 10% to 20% of the adult population [1]. Dry eye disease can affect visual function, and thus common tasks of daily living, such as reading, speed, and driving are adversely affected by this condition. Inflammation has been recognized as an important process in these diseases [1]. Interleukin-1β (IL-1β) is one of potent proinflammatory cytokines implicated in tissue damages. IL-1β acts as a major mediator in the pathogenesis of eye diseases, promoting inflammation, apoptosis, and accumulation of extracellular matrix [1], [2]. Interleukin-1 receptor-1 (IL-1R1)-deficient mice show attenuated production of ocular surface inflammatory cytokines in experimental dry eyes [3].

The implication of IL-1β in inflammatory responses has been shown to be mediated through up-regulation of inflammatory genes, including matrix metalloproteinase-9 (MMP-9) [4]. MMPs are a family of zinc-dependent endopeptidases that primarily degrade components of the extracellular matrix (ECM). Remodeling of the ECM by MMPs is important for both physiological and pathological processes, including organ generation/regeneration, angiogenesis, wound healing, inflammation, and tumor growth [5]–[7]. Moreover, MMP-9 is implicated in a number of pathological conditions, such as eye diseases [8], [9]. Indeed, our previous study indicated that IL-1β can induce MMP-9 expression in A549 cells [4]. However, the mechanisms of IL-1β-mediated MMP-9 expression in Statens Seruminstitut Rabbit Corneal Cells (SIRCs) are unclear. Thus, in this study, we investigated the signaling pathways involved in IL-1β-regulated MMP-9 expression and SIRCs migration.

Mitogen-activated protein kinase (MAPK) signal transduction pathways are ubiquitous and highly evolutionarily conserved mechanisms of eukaryotic cell regulation. The multiple MAPK pathways present in all eukaryotic cells enable coordinated and integrated responses to diverse stimuli, including hormones, growth factors, and cytokines. In mammals, three prominent groups of MAPKs have been identified: p42/p44 MAPK, p38 MAPK, and JNK1/2. Recent evidence suggests the involvement of MAPKs in several pathophysiological processes in retina and cornea [10]–[12]. Moreover, these MAPKs have been shown to be involved in MMP-9 induction in various cell types [5], [6], [13]. Therefore, the role of MAPKs in MMP-9 expression and cell migration induced by IL-1β is still unclear in SIRCs.

The activation of AP-1 and NF-κB transcription factors is critical for a wide range of processes such as immunity, inflammation, cell development, growth, and survival. They are activated by a variety of stimuli including cytokines, ionizing radiation, and oxidative stress. AP-1 transcription factor, a dimeric complex consists of Jun, Fos, Maf, and ATF (activating transcription factor) family DNA-binding proteins [14]. Moreover, NF-κB, c-Jun, and c-Fos have been shown to be involved in inflammatory and apoptotic responses in human corneal epithelial (HCE) cells [10]. In addition, these transcription factors are also required for MMP-9 induction in various cell types [6], [7], [15]. Thus, we investigated the roles of AP-1 and NF-κB in IL-1β-induced MMP-9 expression in SIRCs. On the other hand, MAPKs have been shown to regulate AP-1 and NF-κB activation [6], [16]. The relationship between MAPKs and these transcription factors was investigated in these cells challenged with IL-1β.

In addressing these questions, experiments were undertaken to investigate the mechanisms underlying IL-1β-induced MMP-9 expression in Statens Seruminstitut Rabbit Corneal Cells (SIRCs). These findings suggest that in SIRCs, IL-1β-induced MMP-9 expression was, at least in part, mediated through the p42/p44 MAPK- and JNK1/2-dependent AP-1 and NF-κB pathways.

## Materials and Methods

### Materials

Anti-lamin A, anti-β-actin, anti-p65, anti-p42, anti-JNK2, and anti-IκBα antibodies were from Santa Cruz (Santa Cruz, CA). Anti-phospho-c-Jun, anti-phospho-JNK1/2, anti-phospho-p42/p44 MAPK, and anti-phospho-p38 MAPK antibodies were from Cell Signaling (Danver, MA). Anti-GAPDH antibody was from Biogenesis (New Fields, UK). U0126, SB202190, SP600125, Tanshinone IIA, and Bay11-7082 were from Biomol (Plymouth Meetings, PA). All other reagents were from Sigma (St. Louis, MO). IL-1β was from R&D Systems (Minneapolis, MN).

### Cell culture

The Statens Seruminstitut Rabbit Corneal Cells (SIRCs) were from Bioresource Collection and Research Centre (Hsinchu, Taiwan) and cultured in DMEM/F-12 supplemented with 10% calf serum (CS) and antibiotics (100 U/ml penicillin G, 100 µg/ml streptomycin, and 250 ng/ml fungizone) at 37°C in a humidified 5% CO_2_ atmosphere. When the cultures reached confluence, cells were suspended by 0.05% (w/v) trypsin/1 mM EDTA, and followed to plate onto (1 ml/well) 12-well culture plates and (10 ml/dish) 10-cm culture dishes for the measurement of kinases phosphorylation, protein expression, and mRNA accumulation. In these experiments, IL-1β was added to the serum-free medium and incubated for the indicated time intervals. When the inhibitors were used, they were added 1 h before IL-1β treatment. The concentrations of these inhibitors or vehicle DMSO used lone had no toxic effect or change in the cell viability on SIRCs, excluded by LDH release test or XTT assay (data not shown).

### Transient transfection with siRNAs

Rabbit p42 (AUA UUC UGU CAG GAA CCC UGU GUG A, UCA CAC AGG GUU CCU GAC AGA AUA U, AAA CAA UGU UCU UCC AGU CAA CAG C), JNK2 (ACG UUA CCA GCA GCU GAA ACC AAU U, UUA AGA GGA CAA GUU CAC GAU AAG C, GCU UAU CGU GAA CUU GUC CUC UUA A), and scrambled siRNAs were from Invitrogen (Carlsbad, CA). Transient transfection of siRNAs (100 nM) was performed using a Lipofectamine™ RNAiMAX reagent according to the manufacturer's instructions.

### Cell fractions isolation

Cells were seeded in a 10-cm dish. After cells reached 90% confluence, they were shifted to serum-free DMEM/F-12 medium for 24 h, and then incubated with IL-1β for the indicated time intervals. The cells were washed once with ice-cold PBS, 300 µl of homogenization buffer A (20 mM Tris-HCl, pH 8.0, 10 mM EGTA, 2 mM EDTA, 2 mM dithiothreitol, 1 mM phenylmethylsulfonyl fluoride, 25 µg/ml aprotinin, and 10 µg/ml leupeptin) was added to each dish, and the cells were scraped into a 1.5-ml tube with a rubber policeman. The suspension was sonicated for 5 s at output 1.5 with a sonicator (Misonix, Farmingdale, NY) and centrifuged at 6511×*g* by using the Eppendorf Centrifuge 5810R for 15 min at 4°C. The pellet (nuclear fraction) was re-suspended in 300 µl of homogenization buffer B (1% Triton X-100 in buffer A) and sonicated for 5 s. The supernatant was centrifuged at 19941×*g* for 60 min at 4°C to yield the pellet (membrane fraction) and the supernatant (cytosolic fraction). The membrane fraction was re-suspended in 80 µl of homogenization buffer A. Samples from these supernatant fractions were denatured, subjected to SDS-PAGE, and transferred to nitrocellulose membrane. The translocation of p65 was identified by Western blot analysis using an anti-p65 antibody.

### Western blot analysis

Cells were seeded in 6-well plates. After cells reached 90% confluence, they were shifted to serum-free DMEM/F-12 medium for 24 h, and then incubated with IL-1β for the indicated times. After incubation, cells were rapidly washed with ice-cold PBS, scraped and collected by centrifugation at 1000×*g* for 10 min. The collected cells were lysed with ice-cold lysis buffer containing: 25 mM Tris-HCl, pH 7.4, 25 mM NaCl, 25 mM NaF, 25 mM sodium pyrophosphate, 1 mM sodium vanadate, 2.5 mM EDTA, 2.5 mM EGTA, 0.05% (w/v) Triton X-100, 0.5% (w/v) SDS, 0.5% (w/v) deoxycholate, 0.5% (w/v) NP-40, 5 µg/ml leupeptin, 5 µg/ml aprotinin, and 1 mM phenylmethylsulfonyl fluoride (PMSF). The lysates were centrifuged at 45000×*g* for 1 h at 4°C to yield the whole cell extract. Samples were denatured, subjected to SDS-PAGE using a 10% running gel, and transferred to nitrocellulose membrane. Membranes were incubated with anti-phospho-JNK1/2, anti-phospho-p42/p44 MAPK, or anti-phospho-p38 MAPK antibody for 24 h, and then membranes were incubated with an anti-rabbit or anti-mouse horseradish peroxidase antibody for 1 h. The immunoreactive bands were detected by ECL reagents.

### MMP-9 gelatin zymogram

Cells were plated onto 12-well culture plates and made quiescent at confluence by incubation in serum-free DMEM/F-12 for 24 h and then incubated with IL-1β at 37°C for the indicated time intervals. The culture medium was collected and centrifuged at 10000×*g* for 5 min at 4°C to remove cell debris. The MMP-9 expression was analyzed as previously described [15].

### Immunofluorescence staining

Growth-arrested cells were incubated with IL-1β for the indicated time intervals. After washing twice with ice-cold PBS, cells were fixed, permeabilized, and stained using an anti-p65 antibody as previously described [17]. The images were observed using a fluorescence microscope (Zeiss, Axiovert 200M).

### RT-PCR analysis

Total RNA was isolated with Trizol according to the protocol of the manufacturer. The cDNA obtained from 0.5 µg total RNA was used as a template for PCR amplification as previously described [18]. The primers used were as follows: 5′-TGGCCGGCCACTGTGCGCCCCTCCGAG-3′ (sense) and 5′-CACTAGGTTCACCTCGTTCCGGGTACT-3′ (anti-sense) for MMP-9; 5′-TGACGGGGTCACCCACACTGTGCCCATCTA-3′ (sense) and 5′-CTAGAAGCATTTGCGGTGGACGATG-3′ (anti-sense) for β-actin.

### MMP-9 promoter assay

The rabbit MMP-9 promoter was constructed as previously described [19] with some modifications. The upstream region (−820 to −1) of the rabbit MMP-9 promoter was cloned into the pGL3-basic vector containing the luciferase reporter system. Introduction of a double-point mutation into the NF-κB- or AP-1-binding site to generate pGL-MMP-9-ΔNF-κB, pGL-MMP-9-ΔAP-1, or pGL-MMP-9-ΔdAP-1 was performed using the following (forward) primer:

pGL-MMP-9 wild type:

Sense: 5′-ccccggtaccTGACACCAGCAGGAAGCTGGG-3′ (Kpn1)

Anti-sense: 5′-ccccacgcgtGGTGAGGGGAGCAGCGTCTGGCG-3′ (Mlu1)

pGL-MMP-9-ΔNF-κB (GGAATTCCCC to TTAATTCCCC):

Sense: 5′-TTAATTCCCCAAATCCTGCCTC-3′

Anti-sense: 5′-GGGGAATTAAACCGGGGTAACC-3′

(corresponding to a region from −547 to −539)

pGL-MMP-9-ΔAP-1 (TGAGTCA to TTTGTCA):

Sense: 5′-TTTGTCAGGCACTTGCCTGCAG-3′

Anti-sense: 5′-TGACAAAGGGCCGGTGCAGGGG-3′

(corresponding to a region from −103 to −96)

pGL-MMP-9-ΔdAP-1 (TGAGTCA to TTTGTCA):

Sense: 5′-TTTGTCAGAGGAGGGCTTTCCA-3′

Anti-sense: 5′-TGACAAAGCTTCCTCCTCCCGG-3′

(corresponding to a region from −480 to −474)

The underlined nucleotides indicate the positions of substituted bases. The mutant construct was cloned into the pGL3-basic vector containing the luciferase reporter system. The plasmid was prepared using QIAGEN plasmid DNA preparation kits and transfected into SIRCs using a Lipofectamine reagent (Invitrogen, Carlsbad, CA). To assess the promoter activity, the cells were collected and disrupted by sonication in lysis buffer (25 mM of Tris phosphate, pH 7.8, 2 mM of EDTA, 1% Triton X-100, and 10% glycerol). After centrifugation, luciferase activity in the cell lysates was determined using a luciferase assay system (Promega, Madison, WI). The firefly luciferase activities were standardized for β-galactosidase activity.

### Migration assay

Cells were cultured to confluence in 10-cm dishes and starved with serum-free DMEM/F-12 medium for 24 h. The monolayer cells were scratched manually with a blade to create extended and definite scratches in the center of the dishes with a bright and clear field. The detached cells were removed by washing the cells once with PBS. Serum-free DMEM/F-12 medium with or without IL-1β was added to each dish as indicated after pretreatment with the inhibitors for 1 h, containing a DNA synthesis inhibitor hydroxyurea (10 µM) during the period of incubation. Images of migratory cells from the scratched boundary were observed and acquired at 0 and 24 h with a digital camera and a light microscope (Olympus, Japan). The number of migratory cells was counted from the resulting four phase images for each point and then averaged for each experimental condition. The data presented are generated from three separate assays.

### Cell viability

For measurement of cell viability, cells were seeded into 96-well plates, cultured overnight in DMEM/F-12 medium containing 10% CS, and then incubated with IL-1β (1.5 ng/ml) for 12, 24, or 48 h. Cell viability was determined by MTT assay. Cells treated with medium only served as a negative control group. After removing the supernatant of each well and washing twice by PBS, 20 µl of MTT solution (5 mg/ml in PBS) and 100 µl of medium were then introduced. After incubation for another 4 h, the resultant formazan crystals were dissolved in dimethyl sulfoxide (100 µl) and the absorbance intensity measured by a microplate reader (Bio-RAD 680, CA, USA) at 490 nm with a reference wavelength of 620 nm. All experiments were performed in quadruplicate, and the relative cell viability (%) was expressed as a percentage relative to the untreated control cells.

### Statistical analysis of data

Data were estimated using a GraphPad Prism Program (GraphPad, San Diego, CA). Quantitative data were expressed as mean±S.E.M. and analyzed by one-way ANOVA followed with Tukey's post-hoc test. *P*<0.05 was considered significant.

## Results

### IL-1β induces MMP-9-dependent cell migration

**Figure 1:**
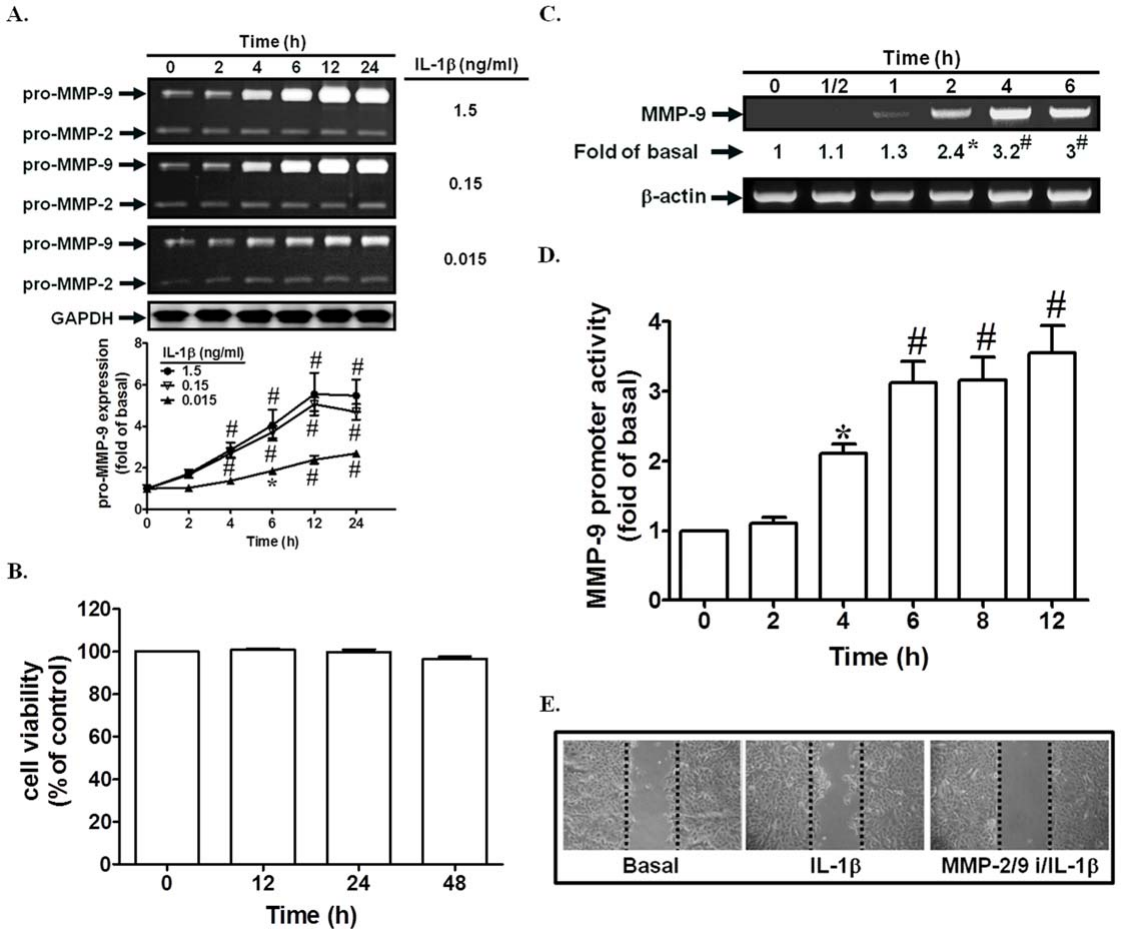
IL-1β induces MMP-9 expression and SIRCs migration. (A) Cells were incubated with IL-1β for the indicated times. The conditioned media were subjected to determine MMP-9 expression by gelatin zymography. The cell lysates were analyzed by Western blot using an anti-GAPDH antibody. (B) Cells were incubated with 1.5 ng/ml IL-1β for the indicated time intervals. The cell viability was measured by MTT assay. (C, D) Cells were incubated with IL-1β for the indicated time intervals. The mRNA levels and promoter activity of MMP-9 were determined by RT-PCR and promoter assay, respectively. (E) Cells were pretreated with 10 µM MMP2/9 inhibitor (MMP2/9 i) for 1 h, and then incubated with IL-1β for 24 h. SIRCs migration was observed. Data are expressed as mean±S.E.M. of three independent experiments. ^*^P<0.05; ^#^ *P*<0.01, as compared with the exposed to vehicle alone.

IL-1β has been shown to induce MMP-9 expression in various cell types [4], [20]. Thus, we investigated whether IL-1β could induce MMP-9 expression in SIRCs. As shown in Fig. 1A, IL-1β markedly induced MMP-9 expression in a time- and dose-dependent manner in these cells. In addition, we used MMT assay to measure the cell viability after IL-1β (1.5 ng/ml) treatment. As shown in Fig. 1B, treatment with IL-1β for 12, 24, or 48 h had no effects on cell viability. On the other hand, we also found that IL-1β enhanced MMP-9 mRNA expression and promoter activity in a time-dependent manner (Figs. 1C and D). MMP-9 has been shown to regulate cell migration in various cell types [4], [7], [20]. In this study, we observed that IL-1β induced SIRCs migration, which was inhibited by the MMP-2/9 inhibitor (Fig. 1E). Taken together, these data suggested that IL-1β could induce MMP-9 expression associated with cell migration.

### IL-1β induces MMP-9 expression via p42/p44 MAPK

**Figure 2:**
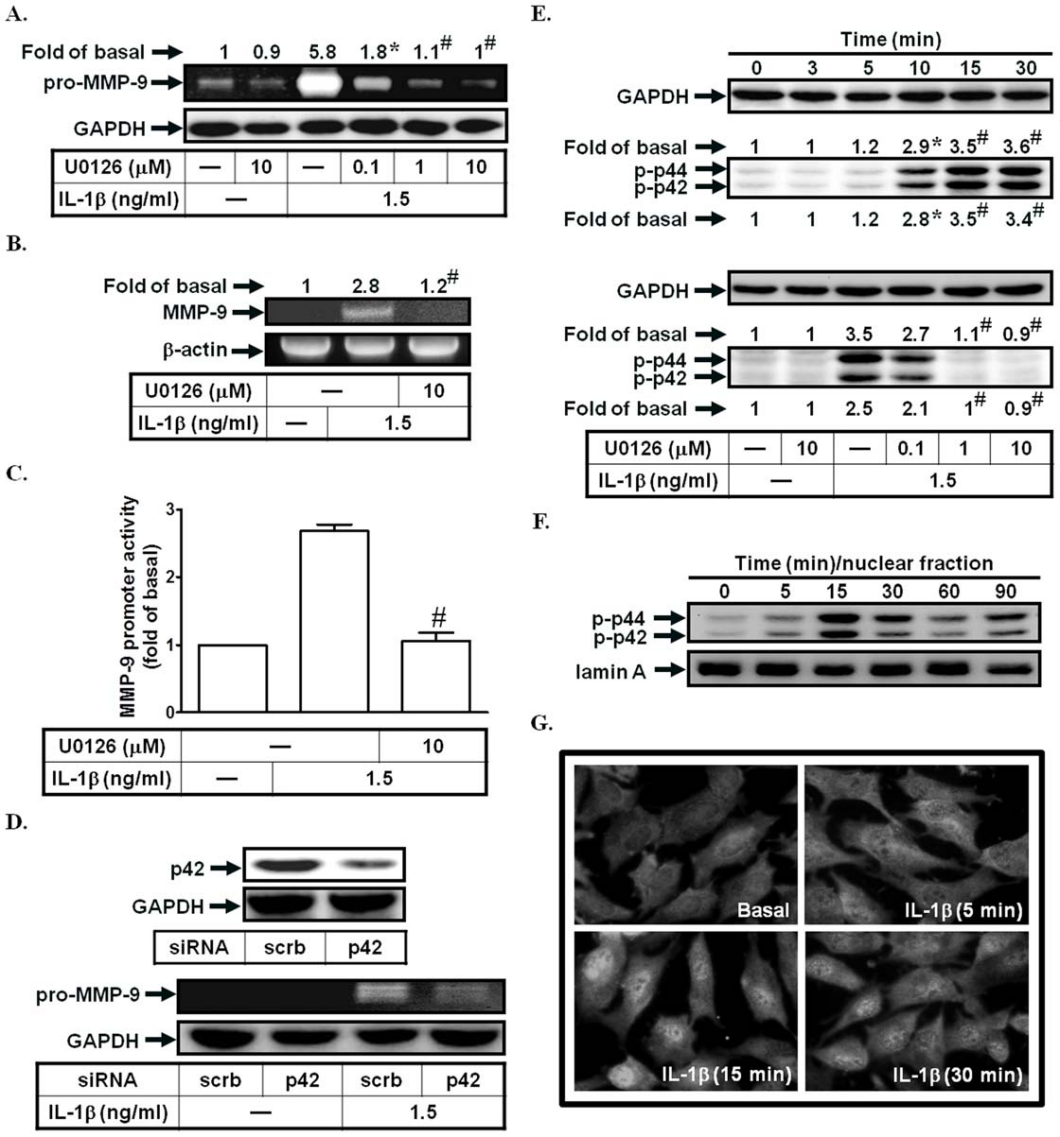
IL-1β induces MMP-9 expression via p42/p44 MAPK. (A) Cells were pretreated with U0126 for 1 h, and then incubated with IL-1β for 12 h. The conditioned media were subjected to determine MMP-9 expression. (B, C) Cells were pretreated with U0126, and then incubated with IL-1β for 4 h (mRNA levels) or 12 h (promoter activity). The mRNA levels and promoter activity of MMP-9 were determined. (D) Cells were transfected with siRNA of scrambled or p42, and then incubated with IL-1β for 12 h. The conditioned media were subjected to determine MMP-9 expression. The protein levels of p42 were determined by Western blotting. (E) Cells were treated with IL-1β for the indicated time intervals or pretreated with U0126, and then treated with IL-1β for 30 min. The phospho-p42/p44 MAPK protein expression was determined by Western blotting. (F, G) Cells were treated with IL-1β for the indicated time intervals. The nuclear fractions were prepared and subjected to Western blotting using an anti-phospho-p42/p44 MAPK antibody. Lamin A was used as a marker protein for nuclear fractions. The translocation of p42/p44 MAPK was observed using a fluorescence microscope. Data are expressed as mean±S.E.M. of three independent experiments. ^*^P<0.05; ^#^ *P*<0.01, as compared with the cells exposed to IL-1β alone [A, B, C, E (lower panel)] or vehicle alone (E, upper panel).

MAPKs integrate signals from numerous receptors and translate these signals into cellular functions. MAPKs are critical for cell metabolism, migration, production of pro-inflammatory mediators, survival and differentiation. Previous study indicated that p42/p44 MAPK is involved in IL-5-induced MMP-9 expression and migration [13]. In this study, we used the MEK1/2 inhibitor (U0126) to investigate the role of p42/p44 MAPK in IL-1β-enhanced MMP-9 expression. As shown in Figs. 2A–C, pretreatment with U0126 markedly inhibited IL-1β-induced MMP-9 expression, mRNA levels, and promoter activity. To confirm the critical role of p42/p44 MAPK in IL-1β-induced MMP-9 expression, p42 siRNA was used. As shown in Fig. 2D, transfection with p42 siRNA inhibited IL-1β-induced MMP-9 expression. On the other hand, IL-1β directly stimulated p42/p44 MAPK phosphorylation in a time-dependent manner, which was reduced by U0126 (Fig. 2E). Extracellular ATP activates nuclear translocation of ERK1/2 leading to the induction of MMPs expression in human endometrial stromal cells [21]. Thus, we also observed whether IL-1β could stimulate p42/p44 MAPK translocation. As shown in Figs. 2F and G, in SIRCs, IL-1β markedly induced p42/p44 MAPK translocation from the cytosol to the nucleus. These data suggested that IL-1β-induced MMP-9 expression is mediated through a p42/p44 MAPK-dependent pathway in SIRCs.

### IL-1β induces MMP-9 expression via a JNK1/2

**Figure 3:**
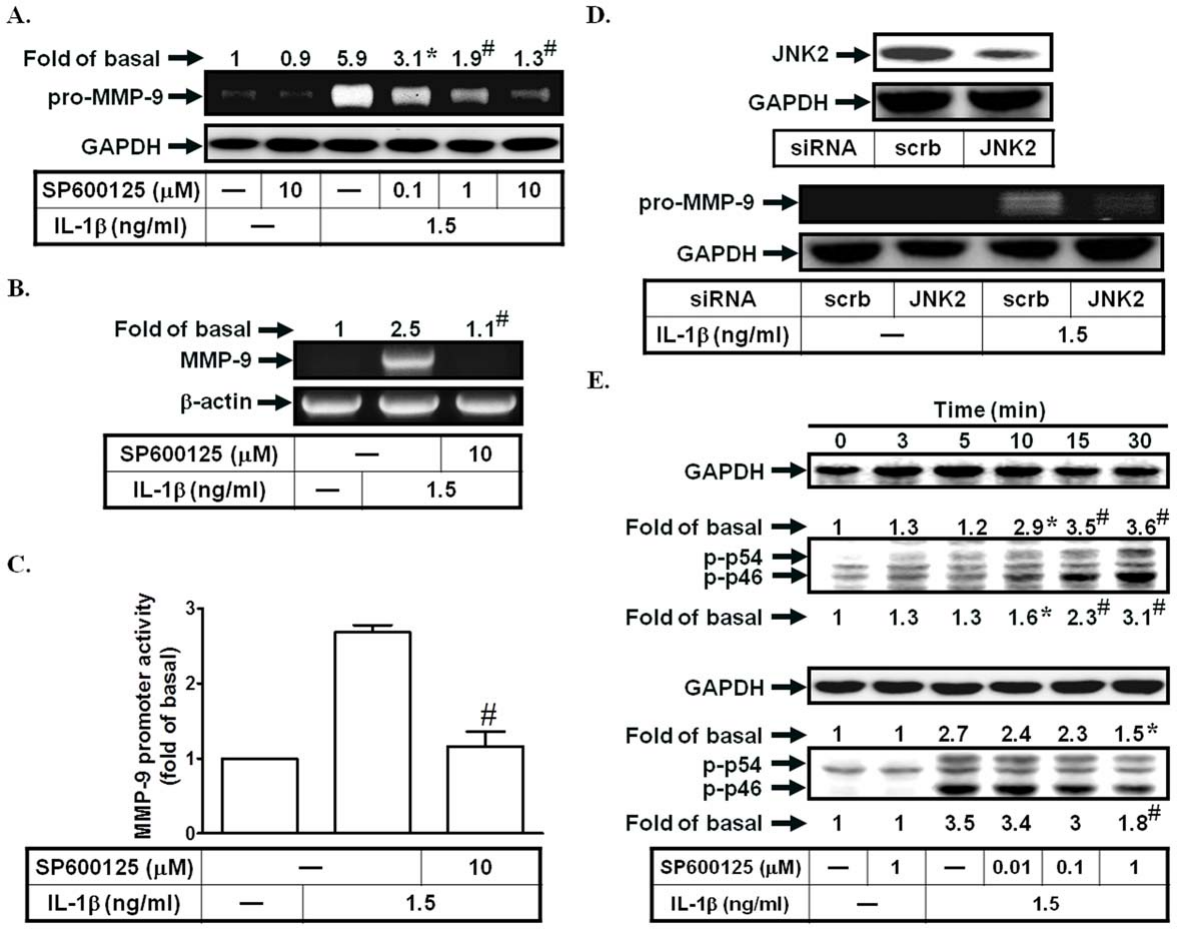
IL-1β induces MMP-9 expression via JNK1/2. (A) Cells were pretreated with SP600125 for 1 h, and then incubated with IL-1β for 12 h. The conditioned media were subjected to determine MMP-9 expression. (B, C) Cells were pretreated with SP600125, and then incubated with IL-1β for 4 h (mRNA levels) or 12 h (promoter activity). The mRNA levels and promoter activity of MMP-9 were determined. (D) Cells were transfected with siRNA of scrambled or JNK2, and then incubated with IL-1β for 12 h. The conditioned media were subjected to determine MMP-9 expression. The protein levels of JNK2 were determined by Western blotting. (E) Cells were treated with IL-1β for the indicated time intervals or pretreated with SP600125, and then treated with IL-1β for 30 min. The phospho-JNK1/2 protein expression was determined by Western blotting. Data are expressed as mean±S.E.M. of three independent experiments. ^*^P<0.05; ^#^ *P*<0.01, as compared with the cells exposed to IL-1β alone [A, B, C, E (lower panel)] or vehicle alone (E, upper panel).

Previous study indicated that JNK1/2 was involved in Moraxella catarrhalis lipooligosaccharide (LOS)-induced MMP-9 expression [22]. Here, we used the JNK1/2 inhibitor (SP600125) to investigate the role of JNK1/2 in IL-1β-enhanced MMP-9 expression. As shown in Figs. 3A–C, pretreatment with SP600125 markedly inhibited IL-1β-induced MMP-9 expression, mRNA levels, and promoter activity. To confirm the critical role of JNK1/2 in IL-1β-induced MMP-9 expression, JNK2 siRNA was used. As shown in Fig. 3D, transfection with JNK2 siRNA inhibited IL-1β-induced MMP-9 expression. On the other hand, IL-1β also directly stimulated JNK1/2 phosphorylation in a time-dependent manner, which was reduced by SP600125 (Fig. 3E). These data suggested that IL-1β-induced MMP-9 expression is mediated through a JNK1/2-dependent pathway in SIRCs.

### p38 MAPK activation is not involved in IL-1β-induced MMP-9 expression in SIRCs

**Figure 4:**
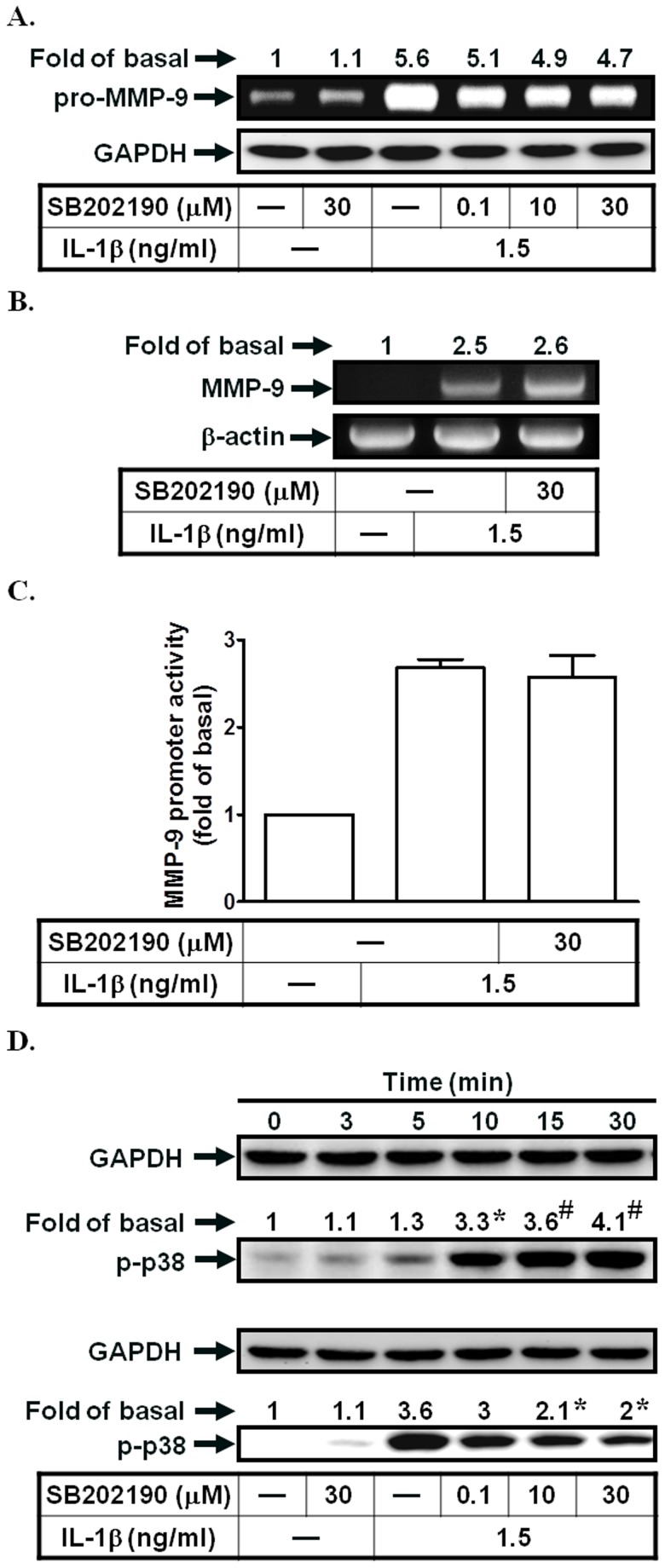
p38 MAPK activation is not involved in IL-1β-induced MMP-9 expression. (A) Cells were pretreated with SB202190 for 1 h, and then incubated with IL-1β for 12 h. The conditioned media were subjected to determine MMP-9 expression. (B, C) Cells were pretreated with SB202190, and then incubated with IL-1β for 4 h (mRNA levels) or 12 h (promoter activity). The mRNA levels and promoter activity of MMP-9 were determined. (D) Cells were treated with IL-1β for the indicated time intervals or pretreated with SB202190, and then treated with IL-1β for 30 min. The phospho-p38 MAPK protein expression was determined by Western blotting. Data are expressed as mean±S.E.M. of three independent experiments. ^*^P<0.05; ^#^ *P*<0.01, as compared with the cells exposed to vehicle alone (D, upper panel) or IL-1β alone (D, lower panel).

p38 MAPK has also been shown to mediate MMP-9 expression [5]. In this study, we used a p38 MAPK inhibitor (SB202190) to investigate its role in IL-1β-enhanced MMP-9 expression. As shown in Figs. 4A–C, pretreatment with SB202190 had no effects on IL-1β-induced MMP-9 expression, mRNA levels, and promoter activity. However, IL-1β directly stimulated p38 MAPK phosphorylation in a time-dependent manner, which was reduced by SB202190 (Fig. 4D). Thus, IL-1β-induced MMP-9 expression may be independent on p38 MAPK phosphorylation in SIRCs.

### AP-1 plays a key role in IL-1β-induced MMP-9 expression

**Figure 5:**
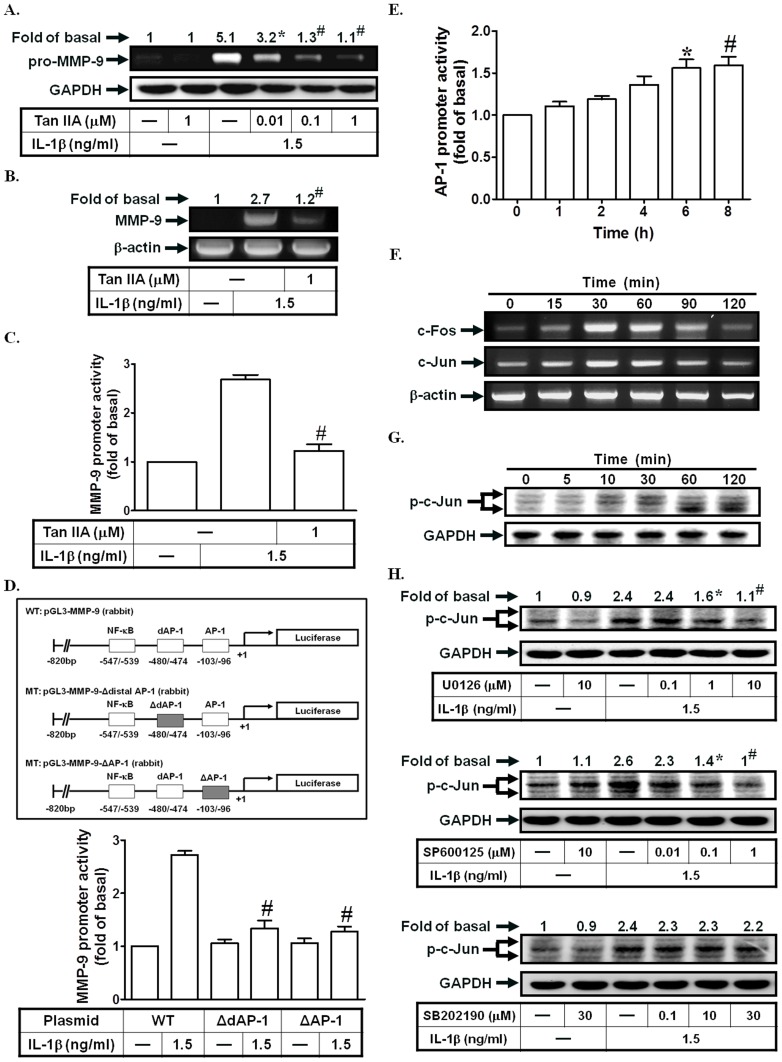
AP-1 is required for IL-1β-induced MMP-9 expression. (A) Cells were pretreated with Tanshinone IIA for 1 h, and then incubated with IL-1β for 12 h. The conditioned media were subjected to determine MMP-9 expression. (B, C) Cells were pretreated with Tanshinone IIA, and then incubated with IL-1β for 4 h (mRNA levels) or 12 h (promoter activity). The mRNA levels and promoter activity of MMP-9 were determined. (D) Cells were transfected with wild-type MMP-9 promoter and AP-1-mutated MMP-9 promoter, and then incubated with IL-1β for 12 h. The promoter activity of MMP-9 was determined. (E) Cells were treated with IL-1β for the indicated time intervals. The AP-1 promoter activity was measured. (F) Cells were treated with IL-1β for the indicated time intervals. The mRNA levels of c-Fos and c-Jun were determined. (G, H) Cells were treated with IL-1β for the indicated time intervals or pretreated with U0126, SP600125, or SB202190, and then treated with IL-1β for 60 min. The levels of c-Jun phosphorylation were analyzed by Western blotting. Data are expressed as mean±S.E.M. of three independent experiments. ^*^P<0.05; ^#^ *P*<0.01, as compared with the cells exposed to IL-1β alone (A, B, C, H), the cells exposed to IL-1β-treated cells transfected with wild-type MMP-9 promoter (D), or the cells exposed to vehicle alone (E).

The promoter region of MMP-9 possesses an AP-1 binding site that is regulated by several external stimuli in different cell types [16]. Here, we used the AP-1 inhibitor (Tanshinone IIA) to investigate its role in IL-1β-enhanced MMP-9 expression. As shown in Figs. 5A–C, pretreatment with Tanshinone IIA markedly inhibited IL-1β-induced MMP-9 expression, mRNA levels, and promoter activity. Next, we used a point-mutated AP-1 MMP-9 promoter construct to confirm the role of AP-1 in IL-1β-mediated MMP-9 promoter induction. As shown in Fig. 5D, IL-1β-stimulated MMP-9 promoter activity was prominently attenuated in SIRCs transfected with the point-mutated AP-1 MMP-9 promoter. On the other hand, we found that IL-β markedly enhanced AP-1 promoter activity and c-Jun/c-Fos mRNA levels (Figs. 5E and F). Finally, we investigated whether IL-1β could stimulate c-Jun phosphorylation. IL-1β stimulated c-Jun activation in a time-dependent manner (Fig. 5G), which was attenuated by U0126 or SP600125, but not by SB202190 (Fig. 5H), suggesting that IL-1β-stimulated c-Jun phosphorylation is mediated through JNK1/2 or p42/p44 MAPK in SIRCs. Thus, IL-1β induced MMP-9 expression via an AP-1-dependent signaling pathway.

### NF-κB is required for IL-1β-induced MMP-9 expression

**Figure 6:**
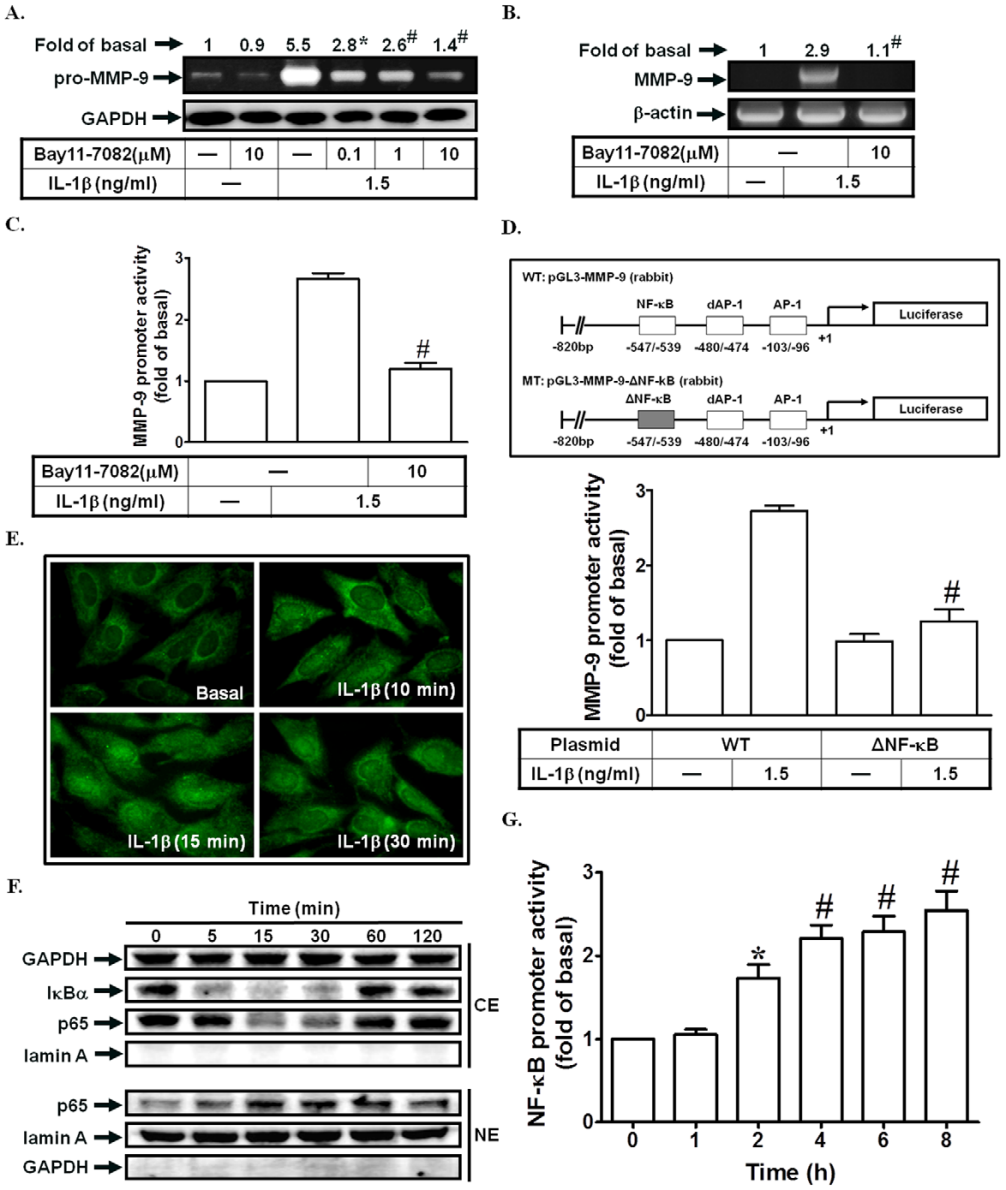
NF-κB is required for IL-1β-induced MMP-9 expression. (A) Cells were pretreated with Bay11-7082 for 1 h, and then incubated with IL-1β for 12 h. The conditioned media were subjected to determine MMP-9 expression. (B, C) Cells were pretreated with Bay11-7082, and then incubated with IL-1β for 4 h (mRNA levels) or 12 h (promoter activity). The mRNA levels and promoter activity of MMP-9 were determined. (D) Cells were transfected with wild-type MMP-9 promoter and NF-κB-mutated MMP-9 promoter, and then incubated with IL-1β for 12 h. The promoter activity of MMP-9 was determined. (E) Cells were treated with IL-1β for the indicated times. The translocation of p65 was observed using a fluorescence microscope. (F) Cells were treated with IL-1β for the indicated times. The nuclear and cytosolic fractions were prepared and subjected to Western blotting using an anti-IκBα or anti-p65 antibody. Lamin A and GAPDH were used as a marker protein for nuclear and cytosolic fractions, respectively. (G) Cells were treated with IL-1β for the indicated times. The NF-κB promoter activity was measured. Data are expressed as mean±S.E.M. of three independent experiments. ^*^P<0.05; ^#^ *P*<0.01, as compared with the cells exposed to IL-1β alone (A–C), the IL-1β-treated cells transfected with wild-type MMP-9 promoter (D), or the cells exposed to vehicle alnoe (G).

NF-κB regulates the expression of a large number of genes involved in inflammation. Moreover, NF-κB has also been shown to regulate MMP-9 induction in various cell types [7], [13], [15]. Here, we found that pretreatment with the inhibitor of NF-κB (Bay11-7082) significantly reduced IL-1β-stimulated MMP-9 expression, mRNA levels, and promoter activity (Figs. 6A–C). Next, we used a point-mutated NF-κB MMP-9 promoter construct to confirm the role of NF-κB in IL-1β-mediated MMP-9 promoter induction. As shown in Fig. 6D, IL-1β-stimulated MMP-9 promoter activity was prominently attenuated in SIRCs transfected with the point-mutated NF-κB MMP-9 promoter. NF-κB is sequested in the cytoplasm associated with IκBα. The IKK is an immediate upstream effector containing either IKKα or IKKβ kinase that phosphorylates IκBα or IκBβ when activated by various stimuli [23]. Phosphorylated IκBs are degraded by the ubiquitin-proteosome and the free NF-κB heterodimer translocates into the nucleus [23]. Thus, we also observed whether IL-1β could stimulate NF-κB p65 subunit translocation in SIRCs. As shown in Figs. 6E and F, IL-1β markedly induced p65 translocation from the cytosol into the nucleus and IκBα degradation. We further showed that IL-1β could increase NF-κB promoter activity (Fig. 6G). Taken together, these data showed that IL-1β-induced MMP-9 expression in mediated through an NF-κB-dependent signaling pathway in SIRCs.

### IL-1β stimulates AP-1 and NF-κB promoter activation leading to cell migration

**Figure 7:**
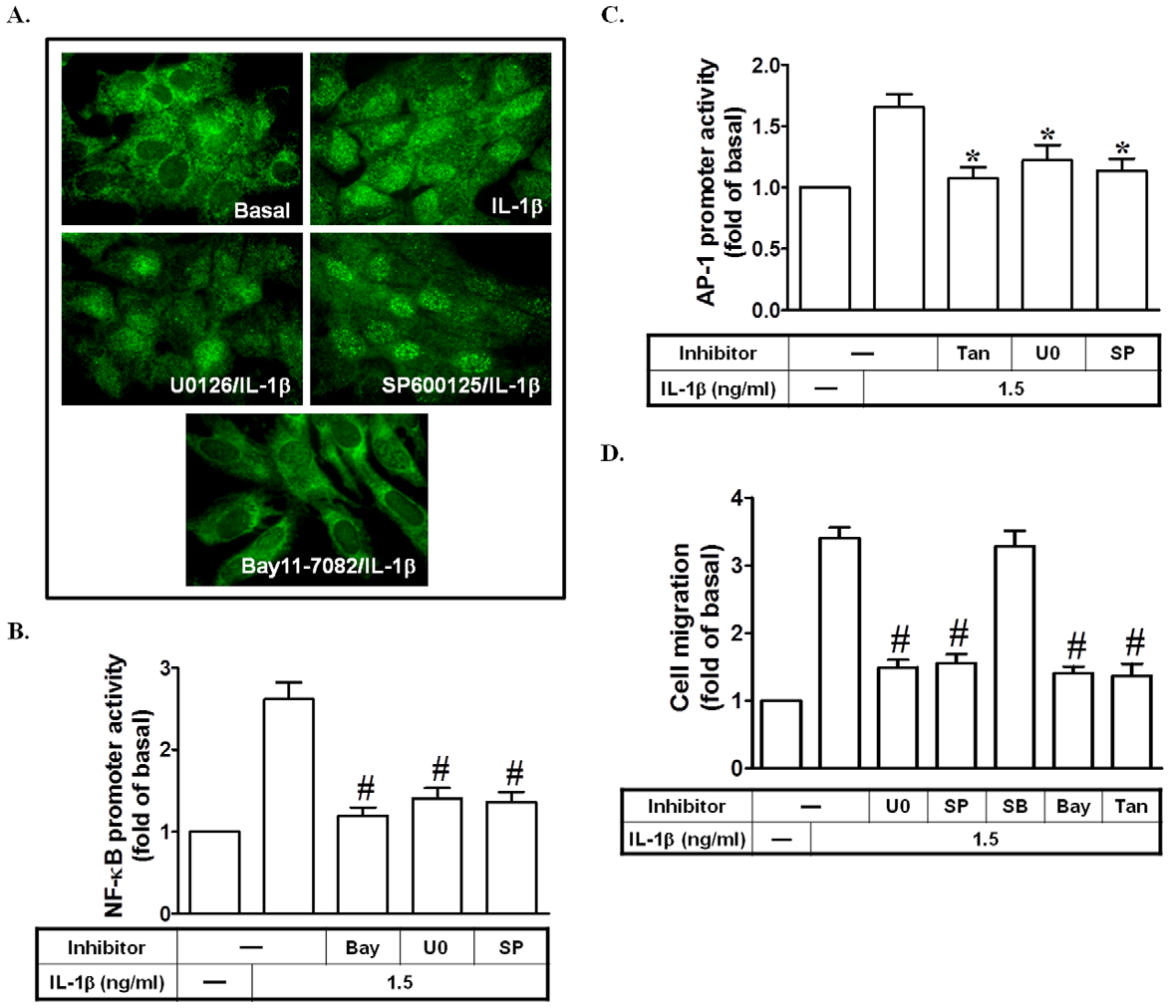
IL-1β stimulates AP-1 and NF-κB promoter activation leading to cell migration. (A) Cells were pretreated with U0126, SP600125, or Bay11-7082 for 1 h, and then treated with IL-1β for 15 min. The translocation of p65 was observed using a fluorescence microscope. (B) Cells were pretreated with Bay11-7082, U0126, or SP600125, and then incubated with IL-1β for 8 h. The NF-κB promoter activity was measured. (C) Cells were pretreated with Tanshinone IIA, U0126, or SP600125 for 1 h, and then incubated with IL-1β for 8 h. The AP-1 promoter activity was measured. (D) Cells were pretreated with Bay11-7082, Tanshinone IIA, SB202190, U0126, or SP600125 for 1 h, and then incubated with IL-1β for 24 h. SIRCs migration was observed. Data are expressed as mean±S.E.M. of three independent experiments. ^*^P<0.05; ^#^ *P*<0.01, as compared with the cells exposed to IL-1β alone.

Since activation of p42/p44 MAPK, JNK1/2, AP-1 and NF-κB was necessary for IL-1β-induced MMP-9 expression in SIRCs, it would be important to differentiate whether phosphorylation of p42/p44 MAPK and JNK1/2 was associated with activation of AP-1 and NF-κB. To examine this possibility, NF-κB p65 translocation, activation of AP-1 and NF-κB promoter activity, and cell migration were assessed by following IL-1β stimulation in the presence of inhibitors for MEK1/2, JNK1/2, AP-1, or NF-κB. As shown in Fig. 7A, IL-1β-induced NF-κB p65 translocation was attenuated by pretreatment with Bay11-7082, but not by U0126 or SP600125 in SIRCs. However, IL-1β-enhanced NF-κB promoter activity was attenuated by pretreatment with Bay11-7082, U0126, or SP600125 (Fig. 7B). We also observed that pretreatment with Tanshinone IIA, U0126, or SP600125 but not SB202190 reduced IL-1β-enhanced AP-1 promoter activity (Fig. 7C). Finally, we established that IL-1β could stimulate SIRCs migration via p42/p44 MAPK, JNK1/2, NF-κB, and AP-1 (Fig. 7D).

## Discussion

**Figure 8:**
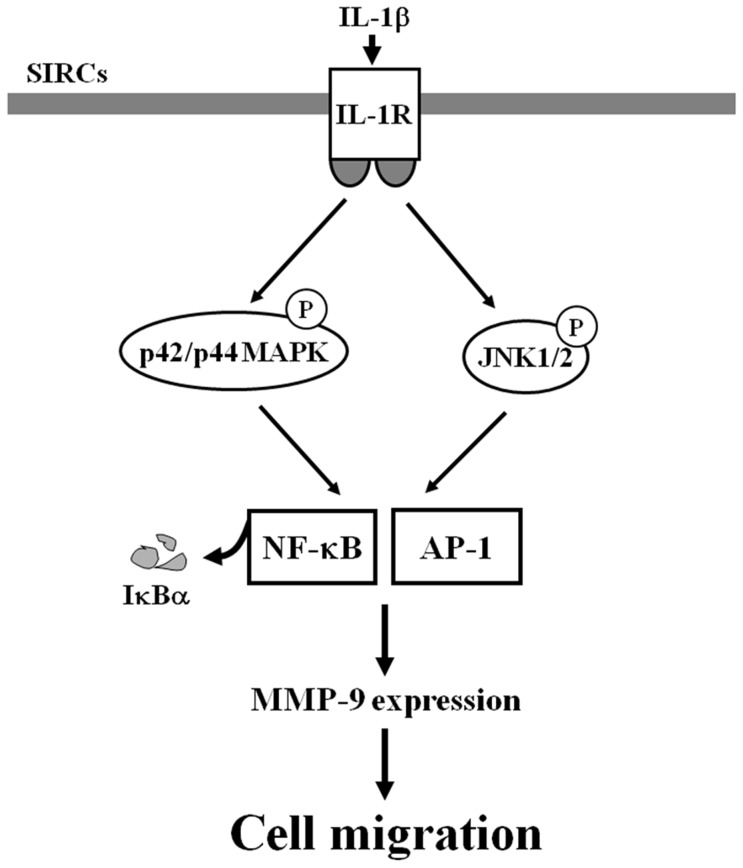
Schematic representation of the signaling pathways involved in the IL-1β-induced MMP-9 expression in SIRCs. IL-1β-induced MMP-9 expression and cell migration are mediated through p42/p44 MAPK- and JNK1/2-dependent AP-1 and NF-κB activation in SIRCs.

Dry eye is a common ocular surface disease, particularly among women and the elderly, with chronic symptoms of eye irritation and, in severe cases, blurred vision. Although the pathogenesis of dry eye disease is not fully understood, it is recognized that inflammation has a prominent role in the development of this debilitating condition. IL-1β acts as a major mediator in the pathogenesis of eye diseases, promoting inflammation, apoptosis, and accumulation of extracellular matrix [1], [2]. The implication of IL-1β in inflammatory responses has been shown to be mediated through up-regulation of inflammatory genes, including MMP-9 [4], [20]. IL-1β has been shown to regulate the activities of MMP-9 through signaling pathways, such as c-Src, MAPKs, AP-1, NF-κB, and p300 in various cells types [4], [20]. However, the mechanisms underlying IL-1β-induced MMP-9 expression in SIRCs remain largely unknown. In this study, we have applied Western blotting, RT-PCR, promoter assay, selective pharmacological inhibitors, and siRNAs to investigate the mechanisms of IL-1β-induced MMP-9 expression in SIRCs. Our results demonstrated that IL-1β-induced MMP-9 expression and SIRCs migration were mediated through the p42/p44 MAPK- and JNK1/2-dependent NF-κB and AP-1 signaling pathways in SIRCs (Fig. 8).

MMPs play a role in pathological processes including inflammation, arthritis, cardiovascular diseases, pulmonary diseases, and cancer. MMP-9 is a secreted multi-domain enzyme that regulates cell-matrix composition. It belongs to the gelatinase subfamily of the MMPs and therefore its main substrate is gelatin (a denatured collagen) [6], [15]. Accumulating evidence demonstrates that IL-1β may activate downstream protein kinases cascades leading to the expression of inflammatory proteins [4], [20]. IL-1α and IL-1β exert similar biological effects, by binding the membrane-bound type I IL-1 receptor (IL-1R1), which then associates with the IL-1-receptor accessory protein (IL-1RAcP) to form a complex that allows intracellular signaling [24]. There is also another type II receptor IL-1R2 which lacks an intracellular-signaling domain, so no downstream signal is initiated when IL-1 binds [24]. Moreover, expression and secretion of MMP-9 are tightly regulated by cytokines, chemokines, eicosanoids, and peptidoglycans [25]. Indeed, in SIRCs, we found that IL-1β could induce MMP-9 mRNA expression and promoter activity. MMP-9 secretion has been shown to regulate cell migration [4]. Here, we observed that pretreatment with MMP2/9 inhibitor could reduce IL-1β-induced SIRCs migration. Thus, we suggested that IL-1β could induce SIRCs migration via MMP-9 induction.

The MAPKs regulate diverse cellular programs by relaying extracellular signals to intracellular responses. In mammals, there are more than a dozen MAPK enzymes that coordinately regulate cell proliferation, differentiation, motility, and survival. The best known are the conventional MAPKs, including the p42/p44 MAPK, JNK1/2, and p38 MAPK [16]. MAPKs also have been shown to regulate MMP-9 induction [16]. Moreover, this is confirmed by our observation that IL-1β-induced MMP-9 expression was reduced by inhibition of JNK1/2 and p42/p44 MAPK. Interestingly, inhibition of p38 MAPK had no effect on IL-1β-induced MMP-9 expression, although IL-1β could stimulate p38 MAPK phosphorylation in SIRCs. Extracellular ATP activates nuclear translocation of ERK1/2 leading to the induction of MMPs expression in human endometrial stromal cells [21]. Moreover, we also found that IL-1β markedly caused p42/p44 MAPK translocation from the cytosol into the nucleus in SIRCs. In the future, we will investigate the detail mechanisms of IL-1β-mediated p42/p44 MAPK activation and translocation associated with genes expression in these cells.

The activation of NF-κB transcription factor is critical for a wide range of processes such as immunity, inflammation, cell development, growth, and survival. It is activated by a variety of stimuli including cytokines, ionizing radiation, and oxidative stress. Thus, we also suggested that IL-1β could induce inflammation via an NF-κB signaling in SIRCs. Indeed, previous study indicated that NF-κB activation plays a key role in MMP-9 induction and cell migration [6], [7], [15]. This is confirmed by our observation that IL-1β-induced MMP-9 expression was reduced via NF-κB inhibition in SIRCs. In response to proinflammatory cytokines, the IκBs are rapidly phosphorylated at two specific serine residues located at their N-terminal region (Ser^32^ and Ser^36^ for IκBα, Ser^19^ and Ser^23^ for IκBβ, Ser^157^ and Ser^161^ for IκBε) and then undergo ubiquitination and proteolysis by the 26S proteasomes, resulting in release and translocation of NF-κB subunits into the nucleus, where it activates transcription of specific target genes [23]. In SIRCs, we also observed that IL-1β could cause NF-κB (p65 subunit) translocation into the nucleus, and then increase MMP-9 gene expression. On the other hand, we established that p42/p44 MAPK and JNK1/2 activation were involved in IL-1β-induced NF-κB promoter activity, but not translocation. Therefore, IL-1β-induced NF-κB activation could promote MMP-9-dependent SIRCs migration and inflammatory responses.

AP-1 is a dimeric transcription factor comprising proteins from several families whose common denominator is the possession of basic leucine zipper (bZIP) domains that are essential for dimerization and DNA binding. Moreover, various stimuli lead to the expression and/or activation of c-Fos and c-Jun products which heterodimerize and bind to AP-1 sites within MMP-9 gene promoters [16]. Recent studies have further demonstrated that several external stimuli can up-regulate MMP-9 expression via AP-1 in different cell types [7], [16]. In SIRCs, IL-1β could increase c-Jun and c-Fos mRNA expression, which may also enhance AP-1 protein expression. Moreover, IL-1β-induced MMP-9 expression was reduced by AP-1 inhibition in these cells. c-Jun is tightly regulated post-translationally and is phosphorylated at two distinct regions. Thus, we observed whether IL-1β could stimulate c-Jun phosphorylation in SIRCs. Indeed, IL-β stimulated c-Jun phosphorylation which was inhibited by U0126 and SP600125. In addition, inhibition of p42/p44 MAPK and JNK1/2 also reduced IL-1β-stimulated AP-1 promoter activity. Therefore, our present study suggested that AP-1 plays a critical role in mediating IL-1β-induced MMP-9 expression leading to cell migration.

In summary, as depicted in Fig. 8, our results showed that in SIRCs, IL-1β stimulated p42/p44 MAPK and JNK1/2 activation, in turn initiates the activation of AP-1 and NF-κB. Activated AP-1 and NF-κB are recruited to the promoter region of MMP-9 leading to an increase of MMP-9 promoter activity and the expression of MMP-9. These results provide new insights into the mechanisms of IL-1β action on SIRCs to regulate the expression of MMP-9 and thus exaggerated the inflammation responses.
