# Correction: IL-1β Promotes Corneal Epithelial Cell Migration by Increasing MMP-9 Expression through NF-κB- and AP-1-Dependent Pathways

**DOI:** 10.1371/annotation/3deca5c8-a7f5-4985-b2eb-c6862ce36e48

**Published:** 2013-12-20

**Authors:** Hui-Ching Tseng, I-Ta Lee, Chih-Chung Lin, Pei-Ling Chi, Shin-Ei Cheng, Ruey-Horng Shih, Li-Der Hsiao, Chuen-Mao Yang

In Figure 3, section D, the Western blot image of GAPDH was posted incorrectly as a duplication from Figure 2, section D. Please view the corrected Figure 3D here: http://plosone.org/corrections/pone.0057955.g003.cn.tif

